# Biomarker changes before and after the 2024 peak burning period in healthy, diabetic, and hypertensive residents of Chiang Mai, Thailand

**DOI:** 10.3389/fpubh.2025.1535448

**Published:** 2025-10-13

**Authors:** Cao Xianfeng, Sumed Yadoung, Phannika Tongchai, Supansa Pata, Woottichai Khamduang, Kriangkrai Chawansuntati, Supachai Yodkeeree, Anurak Wongta, Kanokwan Kulprachakarn, Natthapol Kosashunhanan, Surat Hongsibsong

**Affiliations:** ^1^School of Health Sciences Research, Research Institute for Health Sciences, Chiang Mai University, Chiang Mai, Thailand; ^2^Environmental, Occupational Health Sciences and NCD Research Group, Research Institute for Health Sciences, Chiang Mai University, Chiang Mai, Thailand; ^3^Department of Medical Technology, Faculty of Associated Medical Sciences, Chiang Mai University, Chiang Mai, Thailand; ^4^Research Center for Molecular and Cell Biology, Research Institute for Health Sciences, Chiang Mai University, Chiang Mai, Thailand; ^5^Department of Biochemistry, Faculty of Medicine, Chiang Mai University, Chiang Mai, Thailand

**Keywords:** PAHs, diabetes mellitus, hypertension, lung function biomarkers, PM_2.5_ pollution

## Abstract

**Introduction:**

Burning-related air pollution is a recurrent seasonal problem in Chiang Mai, Thailand, from March to May. Exposure has been linked to pulmonary damage and oxidative stress, measurable via serum Club Cell Protein 16 (CC16) and 8-Iso-prostaglandin F2α (8-iso-PGF2α). Polycyclic aromatic hydrocarbons (PAHs), especially 1-hydroxypyrene (1-OHP), are emitted during incomplete combustion and may contribute to diabetes and hypertension through oxidative pathways. Few studies have examined how PAH exposure from seasonal air pollution affects lung function biomarkers in individuals with these conditions in this region.

**Methods:**

A prospective cohort study was conducted in three Chiang Mai locations, following 127 participants with diabetes and/or hypertension during the before-burning (December 2023) and after-burning (May 2024) periods. Urinary 1-OHP measured PAH exposure, while serum CC16 and 8-iso-PGF2α assessed pulmonary damage and oxidative stress. Structured questionnaires captured participant characteristics and symptoms. Quantitative health-risk assessment (QHRA) converted 1-OHP to benzo[a]pyrene-equivalent doses for estimating lifetime cancer risk (LCR) and hazard quotient (HQ).

**Results:**

From before- to after-burning, cohort-wide means increased significantly: urinary 1-OHP (0.24 ± 0.05 to 0.90 ± 1.21 μmol/mol Cre; *p* < 0.01), serum CC16 (63.15 ± 25.74 to 101.31 ± 48.14 ng/ml; *p* < 0.01), and serum 8-iso-PGF2α (47.44 ± 19.91 to 52.51 ± 20.56 ng/ml; *p* < 0.01). Stratified by comorbidity, hypertensive participants showed a greater 1-OHP increase (0.25 ± 0.35 to 1.31 ± 1.67; *p* < 0.01), while diabetic participants had larger CC16 rises (62.39 ± 18.96 to 93.23 ± 29.92; *p* < 0.01). Among diabetics in the after-burning period, those reporting skin irritation or shortness of breath had lower urinary 1-OHP than asymptomatic peers (0.50 vs. 1.14 and 0.48 vs. 1.08 μmol/mol Cre; *p* = 0.049 and 0.036, respectively). QHRA showed that during the burning season, several age–sex–disease subgroups exceeded the 1 × 10^−^4 LCR benchmark, notably men ≥50 years and some women >60 years, while all HQs remained < 1.

**Conclusion:**

After burning in Chiang Mai substantially increases PAH exposure, pulmonary injury markers, and oxidative stress in individuals with diabetes and hypertension, with differential effects by comorbidity. Cancer risk thresholds were exceeded in older subgroups despite HQs below non-cancer hazard levels, highlighting the need for targeted protection strategies during burn seasons.

## 1 Introduction

Worldwide, environmental pollution, which damaged physical health (PH), could not be ignored (1). Particulate matter (PM_2.5_) was a major content because of the emission of vehicle exhaust and burning crop residues (grain maize, seed maize, and integrated farming) (2). Particulate matter (PM_2.5_) pollution was a major problem in Thailand, Chiangmai, because farmers burn crop residues seasonally from March to May every year in highland agricultural systems (3). Chiangmai was surrounded by mountains, and air pollution from burning crops has caused many health issues in people's lives (4). Polycyclic Aromatic Hydrocarbons (PAHs) were emitted from vehicle exhaust and burning crop residues owing to the incomplete combustion of hydrocarbons. People were exposure to PAHs through air, water, and food (5). Some studies reported that PAHs are a significant potential factor for oxidative stress, inflammatory responses, and lung disease (6). Urinary 1-Hydroxy Pyrene (1-OHP) was an intermediate marker for evaluating PAHs (7).

The inflammatory response played an important role in pulmonary damage and cancer (8). C-reactive protein (CRP) was a marker of inflammation and was produced in the liver (9). The markers of inflammation include Club Cell (farmly Clara cell) protein 16 (CC16). CC16 was a biological marker in the early period of pulmonary epithelial injury and can be detected in the alveoli of the bronchus (10). CC16 also existed in the plasma and spreads in the blood from the alveoli of the bronchus (11).

People could experience oxidative stress when exposed to a burning and smoky environment for an extended period (5). 8-Iso-prostaglandin F2α (8-iso-PGF2α) was a serum marker of oxidative stress and DNA damage (12). It was the gold standard for oxidative stress (13). When the level of oxidative stress was high, 8-iso-PGF2α was produced. It mainly existed in serum, plasma, urine, and other secretions (14).

Some studies reported that pulmonary biomarkers were associated with PM_2.5_. Serum CC16 levels were associated with age, smoking status, and kidney function (15). Exposure to PAHs reduced CC16 levels, and smokers with low levels of CC16 might experience more severe decline in pulmonary function (16). Exposure of alveolar injury to smoke resulted in a temporary increase in serum CC16 levels. When the concentration of 1-OHP increased, the concentration of CC16 decreased (17).

Ros-induced peroxidation of arachidonic acid produced 8-iso-PGF2α. 8-iso-PGF2α was regarded as a biomarker of pulmonary diseases and is involved in the progression of diseases such as chronic obstructive pulmonary disease, interstitial lung disease, acute lung injury, breathing disorders, and lung cancer (18–21). The concentration of 8-iso-PGF2α was high in the obese and smoking groups. However, 8-iso-PGF2α was not associated with asthma (13). As the severity of community-acquired pneumonia increased, the concentration of 8-iso-PGF2α also increased (20).

Diabetes mellitus was a common chronic metabolic disease worldwide (22). It was influenced by life and food habits, including age, smoking, and bad habits, and environmental factors, including noise, air pollution, and electromagnetic fields (23). The study found that PAHs were associated with type 2 diabetes mellitus (T2DM) for reasons including inflammation of active, resistant insulin, change in cytokine release, oxidative stress, and interference of the endocrine system (24). PAHs increased the risk of diabetes development. PAHs increased oxidative stress and secrete endocrine interference of endocrine (25).

PAHs were toxic, mutagenic, and carcinogenic (26). They influenced the function of the cardiovascular system, including blood pressure (BP) (27). One study found that 1-OHP was related to hypertension in adults (27). The study found that PAHs in particulate matter increased diastolic blood pressure and decreased systolic blood pressure (28). The cells in the coronary artery and umbilical vein endothelial cells led to oxidative stress and inflammation caused by PAHs (29). The blood pressure of males increased significantly compared with that of females in response to PAHs (30). However, few studies had focused on lung function and PAHs in participants with diabetes and hypertension. Most studies used only the cross-sectional method, which cannot exclude reversed causation.

Herein, the present study conducted a prospective cohort study to evaluate the relationship between PAHs and lung function biomarkers among participants with diabetes and hypertension in different areas of burning air pollution. This study aimed to evaluate changes in lung function before and after exposure to PAHs among metabolically disordered and oxidate stressed individuals and to formulate a protective measure for the susceptible population.

## 2 Materia and methods

### 2.1 Study design and population

This was a prospective cohort study in Ban Hua Rin, Thung Satok Subdistrict (Place 1), Ban Piang, Ban Mae Subdistrict (Place 2), and Ban Sai Mun, Mae Ka Subdistrict (Place 3), in San Pa Tong District, Chiang Mai Province during December 2023 for visit 1 of before-burning air pollution and May 2024 for visit 2 of after-burning air pollution. The study protocol was approved by the Research Ethics Committee of the Research Institute for Health Science in Chiangmai, Thailand. All procedures conformed to the Declaration of Helsinki (31). The overall sample size of 127 participants was sufficient to detect moderate effect sizes in two-group comparisons (e.g., Cohen's *d* ≈ 0.5) and in correlation analyses (e.g., Spearman's ρ ≈ 0.3) with adequate statistical power (≥80%). However, in subgroup analyses (e.g., stratified by diabetes or hypertension status) and three-group comparisons across the geographic regions, the sample size per group was lower (e.g., *n* = 28 in one region), which might have limited the ability to detect small effect sizes (e.g., *d* < 0.3 or ρ < 0.2).

Participants with diabetes and hypertension were recruited from local clinics in Chiang Mai. To minimize potential confounding effects of medications on biomarker levels, all participants were instructed to withhold their prescribed medications, including antihypertensive agents (e.g., ACE inhibitors, beta-blockers, angiotensin II receptor blockers) and antidiabetic agents (e.g., metformin, insulin), for at least 24 h prior to sample collection. Additionally, participants were advised to avoid strenuous physical activity, alcohol consumption, and foods rich in antioxidants for 12 h before blood and urine sampling. Individuals with poorly controlled diabetes (defined as HbA1c >10%) or those experiencing acute respiratory infections at the time of recruitment were excluded from the study to reduce potential bias.

#### 2.1.1 Data collection

Trained staff collected data from the questionnaire. We used a structured 122-item short-form survey questionnaire (SF-122). The questionnaire included two parts: participant characteristics and physical health (PH). The characteristics of the participants included age, gender identity, occupation, education level, income, weight, height, body mass index, waist circumference, hip circumference, waist-to-hip ratio, waist-to-height ratio, and family members. Physical health (PH) included body temperature, blood oxygen concentration, blood pressure, pulse, respiratory rate, smoking status, alcohol consumption, diabetes, exercise, chronic disease, eye irritation, skin irritation, respiratory irritation, feeling short of breath, feeling dizzy, fainting, losing consciousness, and having pneumonia.

### 2.2 PM_2.5_ measurement

The air quality monitoring stations in the study area were located in three areas: San Pa Tong District, Chiang Mai Province: Ban Hua Rin, Thung Satok Subdistrict, Ban Piang, Ban Mae Subdistrict, and Ban Sai Mun, Mae Ka Subdistrict. The monthly air quality of PM_2.5_, measured between January 2023 and September 2024, was measured and recorded using the Northern Thailand Air Quality Health Index (NTAQHI) (32).

### 2.3 CC16 and 8-iso-PGF2α measurement

Venous blood (3 ml) venous blood was collected by medical laboratory technicians. A 3,000 × g centrifugal machine was used to separate the serum at 4 °C for 15 min, and the serum was stored at −80 °C. We then analyzed the serum CC16 and 8-iso-PGF2α levels. The concentrations of serum CC16 and 8-iso-PGF2α were detected using commercial human CC16 and 8-iso-PGF2α enzyme-linked immunosorbent assay (ELISA) test kits (Bio Aoruida^®^, Guangzhou China) according to the manufacturer's recommendations provided. After the room-temperature equilibrium for 20 min at 25 °C, the detection antibody was added to the microplates and incubated for 1 h. The microplates were read using a microplate reader at a wavelength of 450 nm. Standards and samples were added in duplicate, and the results were calculated for average data. Samples for after- and before-burning air pollution were obtained from the same analytical batch. Intra-assay coefficients of variation for serum CC16 were 5.1%−8%. The range of detectable concentration in serum was 15.1–85.5 pg/ml with *R*^2^ values was 0.982–0.985. Intra-assay coefficients of variation for serum 8-iso-PGF2α were 2.2%−8.3%. The range of detectable concentration in serum was 19.0–68.3 pg/ml with R2 values was 0.982–0.985.

### 2.4 One hydroxy pyrene (1-OHP) measurement

Based on a previous study (78), urinary 1-OHP levels were detected by high-performance liquid chromatography with fluorescent detection (HPLC-FLD). Urine samples from both the after- and before-burning air pollution groups were analyzed. Pool urine samples were analyzed for quality control for each batch of measurements.

### 2.5 Statistical analyses

The standard descriptive statistics were used to show the characteristics of the participants with after- and before-burning air pollution. One-way ANOVA was used to compare age and BMI. The *t*-test was used to determine sex parameters. The normality of the following variables was assessed using the Shapiro-Wilk test for normality, conducted in SPSS version 27: age, sex, smoking history, urinary 1-OHP, serum CC16, serum 8-iso-PGF2α, diabetes status, hypertension status, and PM_2.5_. The Shapiro-Wilk test was selected due to its suitability for medium-sized or smaller samples (*n* ≤ 200) (33). The results indicated that the data did not meet the assumption of normality (*p* < 0.05) for all variables. Normality was further evaluated visually using Q-Q plots and histograms. For comparisons between the after burning and before burning groups, the Mann–Whitney *U* test was used, as it was robust for comparing non-normally distributed continuous variables between two independent groups. For analysis of differences across the three regions, the Kruskal–Wallis *H* test was used, as it was appropriate for comparing non-normally distributed data across three or more independent groups. *Post-hoc* pairwise comparisons were conducted using the Dunn test to identify which specific groups differed significantly (34). Prism was used to show the results in graphs.

### 2.6 Quantitative health risk assessment (QHRA) framework and parameters

We estimated non-cancer hazard quotients (HQ) and lifetime cancer risk (LCR) under the U.S. EPA IRIS/RSL framework using urinary 1-hydroxypyrene (1-OHP) as a biomarker of PAH exposure (35). Creatinine-corrected 1-OHP was translated to BaP-equivalent dose by reverse dosimetry and mixture TEF/RPF conventions (36). LCR was computed as LADD × OSF_BaP and HQ as ADD ÷ RfD_BaP, using IRIS toxicity values (RfD_BaP = 3.0 × 10^−^4 mg/kg·day; OSF_BaP = 2 (mg/kg·day)^−^1; adult-only scenario: 1 (mg/kg·day)^−^1); the legacy OSF = 7.3 (mg/kg·day)^−^1 was examined in sensitivity analysis. As an inhalation cross-check, we considered IUR_BaP = 1 × 10^−^3 (μg/m^3^)^−^1 when air concentrations were available. Interpretation followed commonly used decision ranges (LCR 10^−^6−10^−^4; HQ < 1) (37). Default exposure inputs were IR 20 m^3^/day (sensitivity 18–23), BW 60 kg, EF 350 d/year, ED 30 year, and AT 70 × 365 day (cancer) or ED × 365 days (non-cancer). HQ and LCR were stratified by age ( ≤ 50, 50–60, and >60), sex, and disease status; estimates were deterministic, with uncertainty explored in sensitivity analyses varying k, Pyr:BaP ratios, TEFs/RPFs, and toxicity values.

## 3 Results

### 3.1 The characteristics of participants with diabetes and hypertension in three places

**Figure 1:**
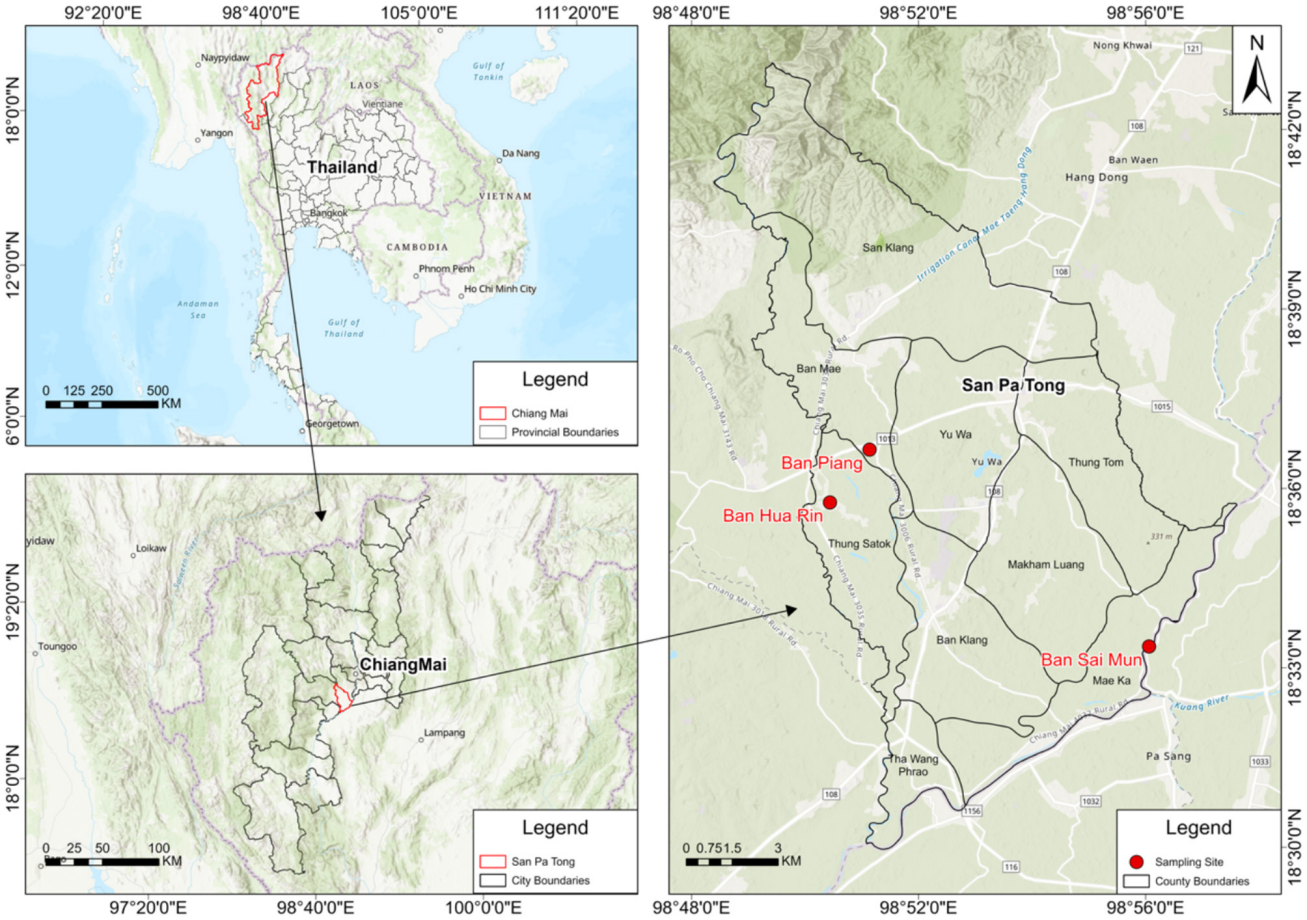
Study area and sampling locations. San Pa Tong District (Chiang Mai, Thailand) with the three sampling communities (Ban Hua Rin, Ban Piang, and Ban Sai Mun) indicated by red markers; administrative boundaries, north arrow, and scale bar are included for reference.

The participants (*N* = 127) were recruited from three locations in San Pa Tong District, Chiang Mai Province: Ban Hua Rin, Thung Satok Subdistrict (Place 1, *N* = 28), Ban Piang, Ban Mae Subdistrict (Place 2, *N* = 50), and Ban Sai Mun, Mae Ka Subdistrict (Place 3, *N* = 49; Figure 1), with a mean age of approximately 60 years. The overall smoking rate was 15.7% (20/127), with a markedly higher proportion of smokers in Place 1 (28.6%) compared to Place 2 (8%), and Place 3 (12.2%). The average body mass index (BMI) was 24.28, and the mean levels of urinary 1-OHP, serum CC16, and serum 8-iso-PGF2α were 0.576 μmol/mol Cre, 81.45 ng/ml, and 49.86 ng/ml, respectively. All three biomarkers were higher in the after-burning season across all areas, with the largest seasonal increases typically in Place 1 (and for CC16 also Place 3); see Table 1 (Δ = after–before burning).

**Table 1:** General characteristics of participants across three study places.

**Variables**	**Place 1 (*N* = 28)**	**Place 2 (*N* = 50)**	**Place 3 (*N* = 49)**	***p*-Value**	**Total (*N* = 127)**
Population characteristics age (years, mean ± SD)	60.81 ± 5.671^a^	59.17 ± 9.395^a^	59.66 ± 7.383^a^	0.79	59.73 ± 7.918
**Sex (** ***n*** **, %)**					
Male	22 (78.6)^a^	33 (66.0)^a^	39 (79.6)^a^	0.939	94 (74.0)
Female	6 (21.4)^a^	17 (34.0)^a^	10 (20.4)^a^		33 (26.0)
**Smoking history (** ***n*** **, %)**					
Yes	8^a^	4^a^	6^a^	0.342	20
No	20^a^	46^a^	43^a^		107
Body mass index (mean ± SD)	23.42 ± 4.12^a^	25.17 ± 12.17^a^	23.87 ± 3.46^a^	0.6	24.28 ± 8.14
PM_2.5_ (μg/m^3^)	24.2^a^	19.52^a^	16.19^b^	< 0.001^**^	19.97
**Urinary 1-OHP (**μ**mol/mol Cre)**					
Before-burning	0.36 ± 0.83^a^	0.14 ± 0.12^a^	0.16 ± 0.20^a^	< 0.01^**^	0.22 ± 0.51
After-burning	1.10 ± 1.67^a^	0.78 ± 0.98^a^	0.72 ± 0.89^a^	0.005^**^	0.88 ± 1.26
**Serum CC16 (ng/ml mean** ±**SD)**					
Before-burning	86.90 ± 41.51^a^	50.48 ± 11.05^b^	62.52 ± 12.61^c^	< 0.001^**^	81.45 ± 42.60
After-burning	142.8 ± 57.36^a^	65.11 ± 30.08^b^	118.13 ± 21.01^c^	< 0.001^**^	101.31 ± 48.14
**Serum 8-iso-PGF2**α **(ng/ml mean** ±**SD)**					
**All**					
Before-burning	53.71 ± 36.92^ac^	41.03 ± 9.13^a^	50.34 ± 10.95^bc^	< 0.001^**^	47.44 ± 19.91
After-burning	63.29 ± 32.93^a^	45.54 ± 15.63^b^	53.73 ± 9.41^c^	< 0.001^**^	52.51 ± 20.56
**Before-burning**					
Smokers	62.87 ± 59.86^a^	48.89 ± 1.96^a^	47.18 ± 7.49^a^	0.977	54.21 ± 34.48
Non-smokers	49.38 ± 20.31^ac^	40.41 ± 9.29^a^	52.33 ± 10.41^bc^	< 0.001^**^	46.40 ± 13.48
**After-burning**					
Smokers	74.65 ± 54.36^ac^	55.32 ± 18.54^a^	50.86 ± 14.66^bc^	0.476	62.73 ± 34.18
Non-smokers	57.62 ± 12.86^ac^	44.67 ± 15.28^a^	54.13 ± 8.68^bc^	< 0.001^**^	50.47 ± 13.86
**Diabetes**					
DM	20	33	34	>0.05	87
Non-DM	8	17	15	>0.05	40
**Hypertension**					
Hypertension	15	33	24	>0.05	72
Non-hypertension	13	17	25	< 0.001^**^	55

Data are presented as mean ± SD or n(%). Statistical comparisons were performed using the Kruskal-Wallis H test for continuous variables and the chi-square or Fisher's exact test for categorical variables. Different superscript letters (a, b, c) in the same row indicate significant differences between locations (*p* ≤ 0.05).

1-OHP, 1-hydroxypyrene; CC16, Clara cell secretory protein 16; 8-iso-PGF2α, 8-iso-prostaglandin F2α; PM_2.5_, particulate matter ≤ 2.5 μm. ^**^Indicates statistical significance at *p* < 0.01.

**Figure 2:**
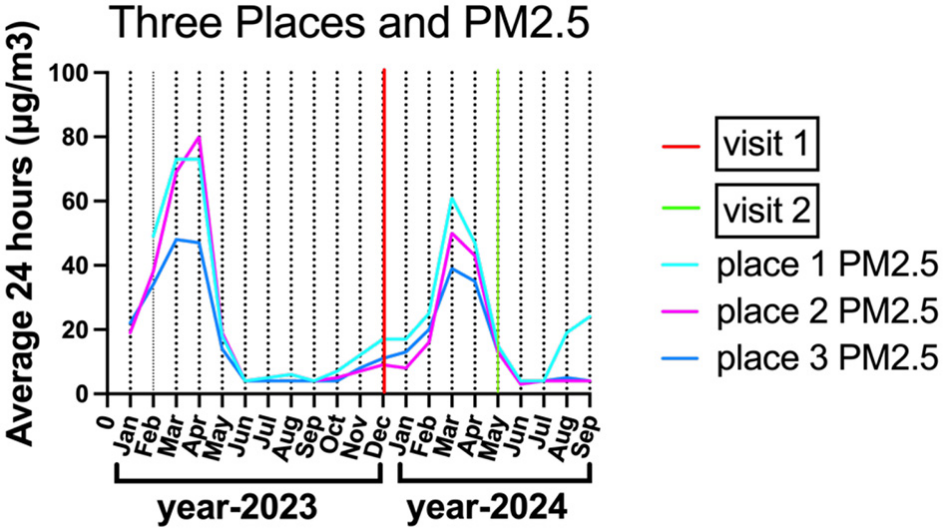
The PM_2.5_ concentrations in three places.

Participants with diabetes (*N* = 87) and hypertension (*N* = 72) were distributed across all three locations. While no significant differences in age, sex, BMI, diabetes, or hypertension were observed among the three places, significant differences were found in urinary 1-OHP (*p* < 0.01), serum CC16 (*p* < 0.001), and serum 8-iso-PGF2α levels (*p* < 0.001). Place 1 exhibited consistently higher PM_2.5_ levels and biomarker concentrations compared to Places 2 and 3 (Table 1; Figure 2).

When stratified by smoking status, non-smokers in Place 1 still exhibited significantly higher serum 8-iso-PGF2α levels than those in Place 2, under both before-burning and after-burning conditions (*p* < 0.001). This trend remained even though PM_2.5_ concentrations were similar between Places 1 and 2. These results suggested that the elevated oxidative stress biomarker levels in Place 1 were not solely attributable to smoking, and other environmental or local factors may be contributing.

### 3.2 Biomarker levels (urinary 1-OHP, serum CC16, and 8-iso-PGF_2_α) in before- and after-burning periods stratified by age and gender

**Figure 3:**
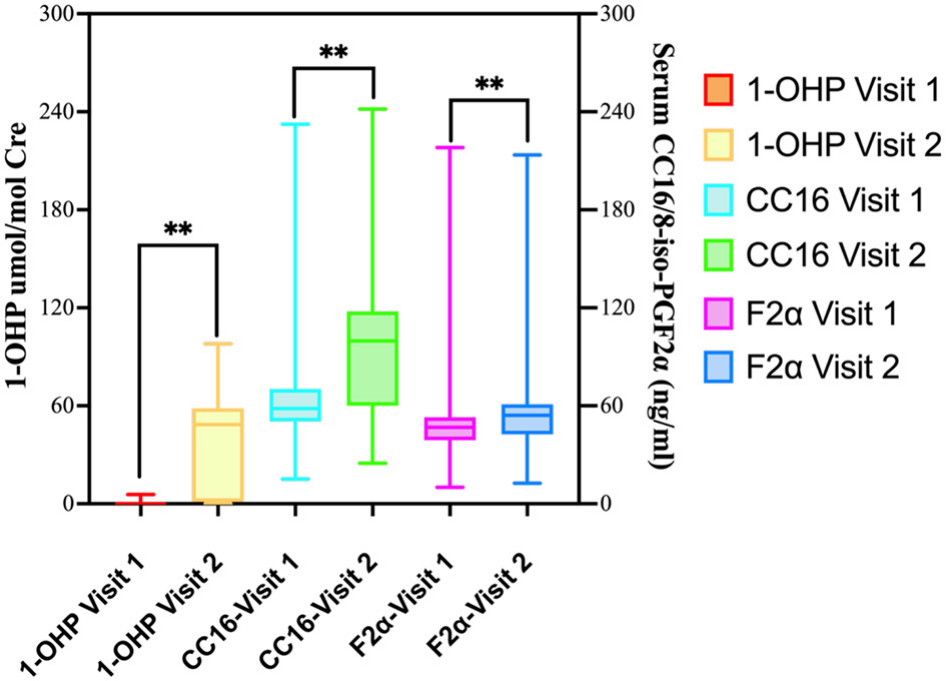
Comparison of oxidative stress biomarkers between before- and After-burning periods.

Compared to before-burning air pollution, the concentrations of serum CC16 and 8-iso-PGF2α increased in after-burning air pollution. There were significant differences (*p* < 0.01) among groups (*p* < 0.01; Table 2). As the concentration of 1-OHP increased, the concentrations of Serum CC16, and Serum 8-iso-PGF2α both increased (Figure 3).

**Table 2:** Urinary 1-OHP, serum CC16, and 8-iso-PGF2α levels under before- and after-burning conditions.

**Biomarker/Variables**	**Before-burning**	**After-burning**	***p* Value**
	**Mean** ±**SD**	**Mean** ±**SD**	
1-OHP (μmol/mol Cre)^a^	0.24 ± 0.05	0.90 ± 1.21	0.01^*^
CC16 (ng/ml)^a^	63.15 ± 25.74	101.31 ± 48.14	0.01^*^
8-iso-PGF2α (ng/ml) ^a^	47.44 ± 19.91	52.51 ± 20.56	0.01^*^

^a^Mann–Whitney U test.

*p value < 0.01.

Table 3 summarizes biomarker changes between before- and after-burning periods stratified by age group and gender. In the overall cohort (*n* = 127), all three biomarkers—urinary 1-OHP, serum CC16, and serum 8-iso-PGF_2_α–increased significantly during the after-burning period (all *p* < 0.001). Across age groups, significant increases in urinary 1-OHP and serum CC16 were observed in all three strata (≤50 years, 50–60 years, and >60 years), whereas serum 8-iso-PGF_2_α showed significant increases in the 50–60 year group (*p* < 0.001) but not in the ≤ 50 year group (*p* = 0.320) and was borderline significant in the >60 year group (*p* = 0.054). Gender-stratified analysis revealed that urinary 1-OHP and serum CC16 increased significantly in both males and females (all *p* < 0.001). Serum 8-iso-PGF_2_α increased significantly in females (*p* < 0.001) but not in males (*p* = 0.153).

**Table 3:** Urinary 1-OHP, serum CC16, and 8-iso-PGF_2_α levels in before- and after-burning periods stratified by age and gender.

**Group**	***n* ^a^**	**1-OHP mean ±SD (μmol/mol Cre) Before to After burning**	***p* ^b^**	**CC16 (ng/ml) mean ±SD Before to After burning**	***p* ^b^**	**8-iso-PGF_2_α (ng/ml) mean ±SD Before to After burning**	***p* ^b^**
Overall	127	0.22 ± 0.51 to 0.88 ± 1.26	< 0.001	81.45 ± 42.60 to 101.31 ± 48.14	< 0.001	47.44 ± 19.91 to 52.51 ± 20.56	< 0.001
Age ≤ 50	21	0.11 ± 0.10 to 0.93 ± 1.61	0.001	59.04 ± 10.91 to 91.41 ± 36.77	0.002	44.21 ± 12.46 to 45.70 ± 11.99	0.32
Age 50–60	59	0.17 ± 0.24 to 0.83 ± 1.17	< 0.001	66.65 ± 35.26 to 105.98 ± 59.39	< 0.001	50.53 ± 27.98 to 56.86 ± 26.85	< 0.001
Age >60	47	0.29 ± 0.70 to 0.91 ± 1.22	< 0.001	61.39 ± 18.73 to 100.04 ± 39.87	< 0.001	46.06 ± 12.44 to 50.65 ± 14.96	0.054
Male	94	0.18 ± 0.26 to 0.95 ± 1.37	< 0.001	64.84 ± 36.66 to 105.93 ± 67.05	< 0.001	51.82 ± 31.65 to 56.03 ± 32.98	0.153
Female	33	0.23 ± 0.57 to 0.86 ± 1.23	< 0.001	62.82 ± 21.20 to 100.12 ± 39.68	< 0.001	46.15 ± 14.01 to 51.31 ± 14.14	< 0.001

^a^n = total number of paired samples included in the Wilcoxon Signed Rank Test (participants with valid measurements for both before- and After-burning periods). The available sample size for individual biomarkers may vary slightly due to missing or insufficient specimens.

^b^p-values are derived from the Wilcoxon Signed Rank Test comparing biomarker levels between before- and After-burning periods.

### 3.3 The characteristics of Urinary 1-OHP, serum CC16, and 8-iso-PGF2α among the diabetes and hypertension participants in the before-burning and after-burning air pollution

Among the participants with diabetes and hypertension, the concentrations of urinary 1-OHP, serum CC16, and 8-iso-PGF2α all increased in the after-burning air pollution group compared to the before-burning air pollution group. There were significant differences in urinary 1-OHP, serum CC16 and 8-iso-PGF2α levels in the before-burning air pollution and after-burning air pollution groups among diabetes and hypertension participants (*p* < 0.01; Table 4).

**Table 4:** Oxidative stress biomarkers stratified by comorbidity (diabetes and hypertension) and pollution level.

	**1-OHP (**μ**mol/mol Cre)**			**CC16 (ng/ml)**			**8-iso-PGF2**α **(ng/ml)**		
**Group/Condition**	**Before-burning mean** ±**SD**	**After-burning mean** ±**SD**	***p*** **Value**	**Before-burning mean** ±**SD**	**After-burning mean** ±**SD**	***p*** **Value**	**Before-burning mean** ±**SD**	**After-burning mean** ±**SD**	***p*** **Value**
Normal (*N* = 80)^a^	0.25 ± 0.64	0.98 ± 1.42	0.01^**^	63.99 ± 29.63	95.27 ± 42.49	0.01^**^	47.74 ± 23.71	54.53 ± 22.85	0.01^**^
NCD-DM (*N* = 37)^a^	0.16 ± 0.22	0.76 ± 1.00	0.01^**^	62.39 ± 18.96	93.23 ± 29.92	0.01^**^	47.82 ± 11.04	48.12 ± 13.68	0.013^*^
Normal (*N* = 66)^a^	0.26 ± 0.86	0.69 ± 0.84	0.028^*^	63.75 ± 20.76	94.58 ± 37.56	0.01^**^	47.65 ± 12.10	53.40 ± 13.03	0.01^**^
Hypertension (*N* = 42)^a^	0.25 ± 0.35	1.31 ± 1.67	0.01^**^	66.75 ± 38.15	99.60 ± 46.56	0.01^**^	49.75 ± 30.63	53.51 ± 30.15	0.01^**^

*p value < 0.05.

**p value < 0.01.

^a^Mann–Whitney U test.

### 3.4 The characteristics of Urinary 1-OHP and different symptoms of participants with diabetes in the after-burning air pollution

In after-burning air pollution, the concentration of urinary 1-OHP increased when participants with Diabetes Mellitus had different symptoms, except for fainting. There were significant differences in the symptoms of skin irritation and shortness of breath (*p* < 0.05). For participants with symptoms of skin irritation and shortness of breath, the level of 1-OHP was lower compared with other symptoms participants (Table 5).

**Table 5:** Urinary 1-OHP levels in diabetic participants with and without self-reported symptoms during After-burning.

**Symptoms**	**1-OHP (**μ**mol/mol Cre) among DM**		***p* Value**
	**After-burning (*****N*** = **46)**		
Eye irritation^a^	Yes (*N* = 35)	0.55 ± 0.53	0.08
	No (*N* = 11)	1.48 ± 1.58	
Skin irritation^a^	Yes (*N* = 26)	0.50 ± 0.43	0.049^*^
	No (*N* = 20)	1.14 ± 1.31	
Respiratory irritation^a^	Yes (*N* = 22)	0.53 ± 0.45	0.095
	No (*N* = 24)	1.00 ± 1.23	
Short of breath^a^	Yes (*N* = 23)	0.48 ± 0.43	0.036^*^
	No (*N* = 23)	1.08 ± 1.23	
Feeling dizzy^a^	Yes (*N* = 14)	0.63 ± 0.62	0.423
	No (*N* = 32)	0.84 ± 1.08	
Fainting^a^	Yes (*N* = 6)	0.83 ± 0.55	0.824
	No (*N* = 40)	0.77 ± 1.01	
Losing consciousness^a^	Yes (*N* = 8)	0.77 ± 0.50	0.957
	No (*N* = 38)	0.80 ± 1.04	
Having pneumonia^a^	Yes (*N* = 18)	0.52 ± 0.46	0.091
	No (*N* = 28)	0.94 ± 1.16	

*p value < 0.05.

^a^Mann–Whitney U test.

### 3.5 Health risk assessment based on urinary 1-OHP

Hazard Quotient (HQ) and lifetime cancer risk (LCR) values were calculated for each age–gender–disease subgroup under both before- and after-burning conditions (Table 6). In the before-burning period, LCR values ranged from 8.01 × 10^−^6 to 5.38 × 10^−^5, all within the acceptable risk range (10^−^6−10^−^4). Corresponding HQ values remained low (0.004–0.024), indicating negligible non-cancer risk. Across age–gender strata, subgroup comparisons showed no statistically significant differences (all *p* > 0.05; *p* ranged approximately 0.078–0.526), where estimable.

**Table 6:** Age- and gender-stratified urinary 1-OHP levels, hazard quotient (HQ), and lifetime cancer risk (LCR) in healthy and comorbidity groups during before- and After-burning periods.

**Age group**	**Gender**	**Healthy controls**	**Hypertension only**	**Diabetes only**	**Both diseases**	***p* (Before burning)**	**Healthy controls**	**Hypertension only**	**Diabetes only**	**Both diseases**	***p* (After burning)**
≤50	Male	1-OHP: 0.06293 ± 0.05099 (*n* = 7); HQ = 0.004; LCR = 9.19E-06; Within	1-OHP: 0.14243 ± 0.10993 (*n* = 9); HQ = 0.009; LCR = 2.08E-05; Within	1-OHP: 0.08700 ± 0.08118 (*n* = 2); HQ = 0.005; LCR = 1.27E-05; Within	1-OHP: 0.08700 ± 0.08118 (*n* = 2); HQ = 0.005; LCR = 1.27E-05; Within	0.380	1-OHP: 1.65233 ± 2.55133 (*n* = 7); HQ = 0.108; LCR = 2.46E-04; Above	1-OHP: 0.64660 ± 0.88530 (*n* = 10); HQ = 0.042; LCR = 9.62E-05; Within	1-OHP: 0.20010 ± 0.12784 (*n* = 2); HQ = 0.013; LCR = 2.98E-05; Within	1-OHP: 0.20010 ± 0.12784 (*n* = 2); HQ = 0.013; LCR = 2.98E-05; Within	0.443
≤50	Female	—	1-OHP: 0.12913 ± 0.12804 (*n* = 4); HQ = 0.008; LCR = 1.88E-05; Within	—	—	—	—	1-OHP: 0.35030 ± 0.13326 (*n* = 4); HQ = 0.023; LCR = 5.21E-05; Within	—	—	—
50–60	Male	1-OHP: 0.25880 ± 0.23171 (*n* = 7); HQ = 0.017; LCR = 3.86E-05; Within	1-OHP: 0.17078 ± 0.27768 (*n* = 37); HQ = 0.011; LCR = 2.55E-05; Within	1-OHP: 0.21800 ± 0.27581 (*n* = 9); HQ = 0.014; LCR = 3.25E-05; Within	1-OHP: 0.21800 ± 0.27581 (*n* = 9); HQ = 0.014; LCR = 3.25E-05; Within	0.436	1-OHP: 1.39323 ± 2.00464 (*n* = 7); HQ = 0.091; LCR = 2.08E-04; Above	1-OHP: 0.70380 ± 1.04818 (*n* = 35); HQ = 0.046; LCR = 1.05E-04; Above	1-OHP: 0.85531 ± 0.86791 (*n* = 9); HQ = 0.056; LCR = 1.27E-04; Above	1-OHP: 0.85531 ± 0.86791 (*n* = 9); HQ = 0.056; LCR = 1.27E-04; Above	0.428
50–60	Female	1-OHP: 0.14720 ± — (*n* = 1); HQ = 0.010; LCR = 2.20E-05; Within	1-OHP: 0.13376 ± 0.10109 (*n* = 14); HQ = 0.009; LCR = 2.00E-05; Within	1-OHP: 0.09340 ± 0.06192 (*n* = 5); HQ = 0.006; LCR = 1.40E-05; Within	1-OHP: 0.09340 ± 0.06192 (*n* = 5); HQ = 0.006; LCR = 1.40E-05; Within	0.317	1-OHP: 0.22850 ± — (*n* = 1); HQ = 0.015; LCR = 3.41E-05; Within	1-OHP: 0.89816 ± 0.96058 (*n* = 14); HQ = 0.059; LCR = 1.35E-04; Above	1-OHP: 0.79572 ± 0.88578 (*n* = 5); HQ = 0.053; LCR = 1.19E-04; Above	1-OHP: 0.79572 ± 0.88578 (*n* = 5); HQ = 0.053; LCR = 1.19E-04; Above	0.841
>60	Male	1-OHP: 0.36048 ± 0.22308 (*n* = 5); HQ = 0.024; LCR = 5.38E-05; Within	1-OHP: 0.31137 ± 0.86665 (*n* = 43); HQ = 0.020; LCR = 4.65E-05; Within	1-OHP: 0.18510 ± 0.23992 (*n* = 24); HQ = 0.012; LCR = 2.76E-05; Within	1-OHP: 0.18003 ± 0.24719 (*n* = 19); HQ = 0.012; LCR = 2.68E-05; Within	0.526	1-OHP: 0.72273 ± 0.83935 (*n* = 4); HQ = 0.047; LCR = 1.06E-04; Above	1-OHP: 0.83084 ± 1.03919 (*n* = 39); HQ = 0.054; LCR = 1.22E-04; Above	1-OHP: 0.87662 ± 1.13954 (*n* = 24); HQ = 0.057; LCR = 1.28E-04; Above	1-OHP: 0.90303 ± 1.23945 (*n* = 19); HQ = 0.059; LCR = 1.31E-04; Above	0.940
>60	Female	1-OHP: 0.05370 ± 0.00042 (*n* = 2); HQ = 0.004; LCR = 8.01E-06; Within	1-OHP: 0.27245 ± 0.40390 (*n* = 13); HQ = 0.018; LCR = 4.07E-05; Within	1-OHP: 0.11001 ± 0.07153 (*n* = 7); HQ = 0.007; LCR = 1.64E-05; Within	1-OHP: 0.12157 ± 0.07084 (*n* = 6); HQ = 0.008; LCR = 1.81E-05; Within	0.078	1-OHP: 1.19490 ± 0.27266 (*n* = 2); HQ = 0.078; LCR = 1.76E-04; Above	1-OHP: 1.29697 ± 2.04367 (*n* = 12); HQ = 0.085; LCR = 1.93E-04; Above	1-OHP: 0.45717 ± 0.41099 (*n* = 7); HQ = 0.030; LCR = 6.74E-05; Within	1-OHP: 0.48962 ± 0.44029 (*n* = 6); HQ = 0.032; LCR = 7.21E-05; Within	0.839

1-OHP = 1-hydroxypyrene concentration (μmol/mol creatinine); HQ, Hazard Quotient; LCR, Lifetime Cancer Risk; Risk level definitions — Within: LCR in 10^−^6−10^−^4 range; Above: LCR > 10^−^4; Below: LCR < 10^−^6 (none in this study). HQ and LCR were calculated according to the USEPA risk assessment guidelines (77).

In the after-burning period, several subgroups exceeded the acceptable upper limit for cancer risk (LCR > 10^−^4). The highest LCR was observed in healthy males aged ≤ 50 years (2.46 × 10^−^4), followed by healthy males aged 50–60 years (2.08 × 10^−^4) and hypertensive males aged 50–60 years (1.05 × 10^−^4). Elevated LCR values were also recorded in diabetic and hypertensive–diabetic subgroups aged 50–60 years and >60 years, ranging from 1.19 × 10^−^4 to 1.93 × 10^−^4. Nevertheless, subgroup comparisons within each age–gender stratum again did not reach statistical significance (all *p* > 0.05; *p* ranged approximately 0.428–0.940). In contrast, HQ values under after-burning conditions remained below 0.11 across all subgroups, suggesting no appreciable non-cancer health risk despite the elevated carcinogenic risk in certain populations. Exceedances (>10^−^4) were concentrated among males aged 50–60 and >60 and, in some categories, females >60, indicating priority groups for mitigation, while the single highest value occurred in healthy males ≤ 50 years.

## 4 Discussion

While this study focused on Chiang Mai, a region with unique geographic and agricultural characteristics (e.g., mountainous terrain and seasonal burning), the findings might not be directly generalizable to other populations or countries with different climate conditions and pollution sources. However, Chiang Mai served as a model for rural-urban transitions in Southeast Asia, and insights from this region could have informed broader environmental health policies in similar contexts.

To the best of our knowledge, the present study was the first two-stage prospective cohort study to investigate the effect of PAHs exposure on lung function in three independent locations in participants with diabetes and hypertension. One study found that the prospective cohort method had benefited from linear regression between independent variables and dependent variables (38). Another study found that the limitations of the prospective cohort method were the high cost and long-time cost (39). In this study, we found that the Mann–Whitney *U* test indicated that the concentrations of urinary 1-OHP, Serum CC16, and Serum 8-iso-PGF2α all increased, and there were significant differences (*p* < 0.01) in the period of after-burning air pollution compared with before-burning air pollution. Previous studies showed that in a short period of after-burning air pollution, the concentrations of urinary 1-OHP, Serum CC16 and Serum 8-iso- PGF2α increased (40–42).

Notably, age modifies the CC16 response: during after-burning pollution, CC16 averaged 10.01 ± 6.91 ng/ml in adults ≥60 years vs. 5.33 ± 4.24 ng/ml in those < 60 years (*p* < 0.05) (43). This pattern is supported by external evidence showing that urinary Σ7OH-PAHs positively correlate with 8-OHdG and 8-iso-PGF2α (*r* = 0.64 and 0.69; *p* < 0.01) (44). CC16 was the gold standard biomarker for lung function (45). Serum 8-iso- PGF2α was the gold standard biomarker of oxidative stress (46). The reason for the increase in CC16 levels was mainly that short-term exposure to particulate air pollution might have damaged the integrity of the lung epithelium and increased epithelial barrier permeability (Aghapour et al. 2022). Serum 8-iso-PGF2α was abundant because of an imbalance in redox homeostasis and reactive oxygen species (ROS) (47).

Among the participants with diabetes, 1-OHP levels all increased compared with before-burning air pollution and after-burning air pollution. One study found that exposure to PAHs accelerated the generation of reactive oxygen species (ROS), which attacked DNA, proteins, and lipids with adverse results (48). Inflammation was caused by ROS in response to activated redox-sensitive transcription factors (49). Continuous inflammation and oxidative stress caused chronic diseases, such as diabetes (50). Another study found that diabetes patients with short-time PAHs were influenced by oxidative stress (23). In this study, we found that compared to participants without hypertension or hypertension, the concentration of 1-OHP increased among hypertensive participants with after-burning air pollution compared to those with before-burning air pollution. One study reported that PAHs increased the risk of oxidative stress and inflammation in the cardiovascular system of hypertensive patients (6). We also found that the 1-OHP concentration of diabetic participants compared to non-diabetic participants decreased in the before-burning or after-burning air pollution groups. One study reported that anti-inflammatory medicines taken for diabetes led to a negative relationship between PAHs and the level of type 2 diabetes in individuals (51). Among coke-oven workers, higher urinary 4-hydroxyphenanthrene (4-OHPh) concentrations were significantly associated with increased diabetes risk (24). Individuals in the highest quartile of total urinary PAHs had higher odds of diabetes (OR 1.56, 95% CI 1.15–2.12; *p* = 0.005), and highest vs. lowest quartile of 2-hydroxynaphthalene was associated with stroke (OR 2.23, 95% CI 1.17–4.25; *p* = 0.016) and diabetes (OR 1.40, 95% CI 1.07–1.82; *p* = 0.015) (52).

In the study, we found that diabetes participants with skin irritation and shortness of breath symptoms were less exposed to PAHs. One study reported that diabetes people with skin irritation were inclined to have problems with infections like fungal and bacterial infections and dry skin (53). Another study found that exposure to air pollution exacerbated skin irritation for diabetes patients due to sensitivity to pollution (54). The other study reported that diabetes people had serious complications such as cardiovascular, obesity, and respiratory diseases. Diabetes people with the symptoms of shortness of breath had a higher risk when they were exposed to PAHs (55). Highest- vs. lowest-quartile exposure was associated with obesity for both urinary 2- (OR 1.46, 95% CI 1.13–1.87) and the sum of PAH metabolites (OR 1.45, 95% CI 1.13–1.87) (25).

Typically, symptoms such as skin irritation and shortness of breath were expected to occur in individuals exposed to after-burning pollution levels. However, our study found that participants with these symptoms had lower levels of 1-OHP, a biomarker for PAH exposure. This counterintuitive result was explained by behavioral modifications, such as staying indoors or using protective gear during after-burning pollution periods, which might have reduced actual pollutant exposure (56). Another possibility was that individuals with these symptoms might have exhibited altered metabolic or excretory responses to pollutants, leading to lower levels of 1-OHP despite experiencing symptoms (57). It was also possible that these participants had developed some level of tolerance or adaptation to the environmental pollution, resulting in a reduced exposure-response effect (58). Further investigation was needed to clarify whether these symptoms reflect increased sensitivity to pollutants or altered exposure-response mechanisms, including potential long-term adaptations to pollution (59).

Polycyclic aromatic hydrocarbons (PAHs) influenced oxidative stress through several pathways, primarily by being metabolized by cytochrome P450 enzymes into reactive intermediates. These intermediates generated reactive oxygen species (ROS), leading to lipid peroxidation, DNA damage, and activation of inflammatory signaling pathways (60). In individuals with diabetes and hypertension, these pathways were exacerbated due to pre-existing redox imbalances and endothelial dysfunction (61). In particular, PAHs might have worsened endothelial cell damage, contributing to impaired vasodilation, increased vascular permeability, and exacerbated blood pressure regulation (29).

The inflammatory response in these individuals might have been further amplified by PAHs, as they could have activated pathways such as NF-κB and MAPK, which were involved in chronic inflammation (62). This persistent inflammation contributed to the development of complications such as cardiovascular diseases (63). Moreover, biomarkers like CC16, secreted in response to epithelial damage, and 8-iso-PGF2α, which reflects oxidative lipid injury, were particularly sensitive to these pollutant-induced oxidative stress pathways (64). Both of these markers were elevated in individuals with pre-existing inflammatory conditions, and their levels were further amplified by systemic inflammation seen in metabolic disorders. However, further studies were needed to explore the detailed molecular mechanisms underlying these processes and their long-term implications for disease progression in polluted environments.

During the after-burning period, our data show a clear divergence between carcinogenic and non-cancer endpoints: several age–sex–disease subgroups exceeded the conventional lifetime cancer risk (LCR) benchmark of 1 × 10^−^4, whereas hazard quotients (HQs) remained well-below 1 (all < 0.11). This pattern was biologically and regulatorily coherent because LCR reflects a non-threshold, cumulative mechanism for PAH carcinogenicity (65), while HQ was a threshold-based construct that flags concern only when equivalent doses approach or surpass the reference dose (66). Exceedances clustered among men ≥50 years and, in some strata, women >60 years; the single highest estimate was in healthy men ≤ 50 years. Taken together, these patterns indicated that exposure intensity and behavior—rather than baseline comorbidity—are the primary risks (67). Although differences across disease categories within each age–sex stratum were not statistically significant (all *p* > 0.05), non-significance was not evidence of no difference (68). Because risk management was anchored to acceptability thresholds, subgroup LCR values exceeding 1 × 10^−^4 warrant mitigation measures (69). In practice, risk management should focus on high-burn days (70). Priority measures included targeted advisories; promoting N95/FFP2 respirators outdoors; using HEPA filtration indoors (71); limiting vigorous outdoor activity; and avoiding grilling/charred foods and tobacco smoke (72). Public health messaging should be tailored to older adults and men, while also addressing younger men experiencing high exposure (73).

One limitation of this study was the lack of data on medication use, such as antidiabetics, antihypertensives, and corticosteroids, which might have influenced biomarkers like CC16 and 8-iso-PGF2α due to their effects on inflammatory and oxidative processes (74). These medications could have acted as confounding factors, potentially impacting observed associations with air pollution. In addition, environmental variables like temperature and humidity, which varied seasonally in Chiang Mai, might influenced pollutant levels and participants' physiological responses (75). Future studies should account for both medication use and seasonal environmental factors to improve the accuracy and generalizability of findings. Beyond medication use and meteorology, our translation from urinary 1-OHP to mixture-level risk assumes stable PAH composition and extrapolates short-term peaks; thus, LCR exceedances are best interpreted as upper bounds (76). Creatinine-only correction and unmeasured behaviors/co-exposures may influence subgroup patterns, while parameter/route choices were not explored probabilistically. Limited stratum sizes, lack of concurrent PAH speciation, and the after-burning context may constrain precision and generalizability.

## 5 Conclusions

Seasonal burning in Chiang Mai is linked to higher PAH exposure and early evidence of airway injury and oxidative stress, with CC16 and 8-iso-PGF2α serving as sensitive indicators. Quantitative risk assessment based on urinary 1-OHP showed that during the high-burn period several age–sex–disease strata exceeded the conventional lifetime cancer-risk benchmark (while all hazard quotients remained < 1). In practice, burn-season measures should prioritize high-risk subgroups with targeted alerts, consistent N95/FFP2 use outdoors, indoor HEPA filtration, and integration of seasonal 1-OHP with CC16/8-iso-PGF2α checks into diabetes and hypertension care. At the policy level, authorities should replace open burning with supported residue-management programs, provide clean-air rooms or purifier subsidies in heavily affected areas, and tailor risk communication to high-risk groups. Future work should link these biomarkers to clinical endpoints and sharpen exposure assessment through PAH speciation and personal monitoring.

## Data Availability

The raw data supporting the conclusions of this article will be made available by the authors, without undue reservation.
